# Global genomic and antimicrobial resistance profiling of *Neisseria gonorrhoeae*: Insights from whole genome sequencing and minimum inhibitory concentration analysis

**DOI:** 10.1371/journal.pntd.0013505

**Published:** 2025-10-06

**Authors:** Elaheh Ebrahimi, Zahra Hadi, Sara Farsioo, Bita Hasani, Farzad Badmasti, Masoumeh Beig, Mohammad Sholeh

**Affiliations:** 1 Department of Microbiology, School of Medicine, Tehran University of Medical Sciences, Tehran, Iran; 2 Department of Microbiology, School of Basic Sciences, Islamic Azad University, Karaj Branch, Karaj, Iran; 3 Department of Microbiology, Faculty of Engineering, North Tehran Branch, Islamic Azad University, Tehran, Iran; 4 Department of Microbiology, Islamic Azad University, Tonekabon, Iran; 5 Department of Bacteriology, Pasteur Institute of Iran, Tehran, Iran; Pontificia Universidad Catolica de Chile, CHILE

## Abstract

**Background:**

The rising antimicrobial resistance (AMR) of *Neisseria gonorrhoeae* is a major global health concern that limits treatment options and complicates disease management. Efflux pump systems and resistance genes are key to bacteria’s ability to evade antibiotics. This study examined the genetic and phenotypic resistance landscape using a large dataset of whole-genome sequences to identify key resistance mechanisms, assess efflux pump gene prevalence, and analyze regional variations in Minimum Inhibitory Concentration (MIC) values to inform treatment strategies and public health interventions.

**Methods:**

A total of 38,585 whole-genome sequences of *N. gonorrhoeae* were analyzed to identify AMR determinants. This study focused on the presence and distribution of efflux pump genes (*mtrC*, *farB*, *norM*, and *mtrA*) and specific resistance genes, including *tet*(C) (tetracycline resistance) and **a*ph*(*3’*)-*I*a** (aminoglycoside resistance). The MIC values were assessed for multiple antibiotics to evaluate resistance trends and regional variations, including penicillin, spectinomycin, zoliflodacin, gentamicin, and fluoroquinolones.

**Results:**

This analysis revealed widespread resistance to multiple antibiotics. Efflux pump genes (*mtrC*, *farB*, *norM*, and *mtrA*) were found in nearly all isolates, highlighting their essential roles in resistance and adaptation. The presence of *tet*(C) and **a*ph* (*3’*)-*Ia* varied across different Gene Presence Patterns, suggesting that regional or therapeutic factors may influence tetracycline and aminoglycoside resistance. High MIC values for penicillin were observed, likely because of *bla*_TEM_, a beta-lactamase gene responsible for beta-lactam resistance. Resistance to spectinomycin is also widespread, raising concerns about the diminishing efficacy of this antibiotic. In contrast, zoliflodacin, gentamicin, and fluoroquinolones exhibited relatively low MIC values, indicating their sustained effectiveness against *N. gonorrhoeae*.

**Discussion:**

Efflux pump systems are key to *N. gonorrhoeae* resistance and adaptability. Regional MIC variations indicate that local antibiotic use shapes resistance patterns. The high resistance to penicillin and spectinomycin highlights the need for alternative treatments, whereas zoliflodacin and fluoroquinolones remain effective but require monitoring. This study emphasizes global AMR surveillance, novel therapies, and targeted antimicrobial stewardship to address multidrug-resistant infections.

## Author summary

*Neisseria gonorrhoeae*, the bacterium responsible for the sexually transmitted infection gonorrhea, has become increasingly resistant to many antibiotics once relied upon for treatment. In this study, we analyzed the complete genetic blueprints of nearly 39,000 *N. gonorrhoeae* samples collected worldwide over the past three decades. We identified key resistance genes, including molecular “pumps” that eject antibiotics from bacterial cells and enzymes that break down drugs like penicillin. By comparing these genetic markers with laboratory measurements of how much antibiotic is needed to stop growth, we revealed that resistance to older drugs such as penicillin and spectinomycin is now widespread. In contrast, newer therapies—like zoliflodacin and gentamicin—remain largely effective, although close monitoring is essential to detect emerging resistance. We also mapped how different bacterial lineages have spread across regions and time, highlighting the global nature of this public health challenge. Our findings underscore the need for coordinated surveillance, smarter antibiotic use, and the development of alternative treatments to stay ahead of drug-resistant gonorrhea.

## 1. Introduction

The antimicrobial resistance (AMR) of *Neisseria gonorrhoeae*, the causative agent of gonorrhea, has become a critical global health concern. Historically, antibiotics such as penicillin and tetracycline were highly effective in treating gonorrhea. However, the emergence of resistant strains has significantly reduced their efficacy, contributing to an escalating public health crisis [1]. The World Health Organization (WHO) and the Centers for Disease Control and Prevention (CDC) have classified *N. gonorrhoeae* as a high-priority pathogen owing to its increasing resistance to third-generation cephalosporins and fluoroquinolones, including ceftriaxone, which is currently considered a last-resort antibiotic for gonorrhea treatment [2]. This resistance has led to treatment failures in several regions worldwide, underscoring the urgent need for continuous surveillance and research on resistance mechanisms to inform effective therapeutic strategies [2].

Advancements in genomic studies, particularly whole-genome sequencing (WGS), have provided critical insights into the molecular mechanisms underlying AMR in *N. gonorrhoeae*. WGS has revealed extensive genetic diversity among resistant strains and has identified specific mutations associated with resistance phenotypes. For example, mosaic *PenA* genes from interspecies recombination have been linked to cephalosporin resistance [3]. Additionally, integrating WGS with antimicrobial susceptibility testing (AST) has improved our understanding of resistance patterns and enhanced the prediction of treatment outcomes [4]. This genomic-phenotypic correlation is invaluable for clinical decision-making, guiding optimal treatment strategies, and strengthening public health responses [4].

Global surveillance programs like the Gonococcal Antimicrobial Surveillance Programme (GASP) and the CDC’s AR Laboratory Network are crucial for monitoring *N. gonorrhoeae* resistance trends [5,6]. However, limited genomic data, high WGS costs, and poor antibiotic stewardship hinder efforts, especially in resource-limited settings [2]. Strengthening surveillance, rapid diagnostics, and stewardship programs is essential [2]. Research on *N. gonorrhoeae* genomics is vital because commensal *Neisseria* may serve as a resistance reservoir [7]. Developing novel antibiotics and combination therapies is the key to combating multidrug resistance (MDR) [8]. Integrating genomic epidemiology with clinical data enhances resistance tracking, enabling targeted interventions and improving treatment strategies [9].

This study leveraged a global collection of whole‐genome sequences to (1) catalog known and novel AMR determinants—including efflux pumps—and correlate these genotypes with phenotypic MIC data; (2) define population structure and evolutionary relationships via MLST and phylogenetic analyses; and (3) map temporal and geographic trends in resistance gene dissemination. By integrating these genomic and phenotypic layers, we aim to inform more effective surveillance frameworks, guide antibiotic stewardship, and support the development of targeted interventions against multidrug‐resistant *N. gonorrhoeae*.

## 2. Methods

### 2.1. Genomic data acquisition and quality control

WGS data for *N. gonorrhoeae* isolates were obtained from the National Center for Biotechnology Information (NCBI) BioSample database (https://www.ncbi.nlm.nih.gov/genbank/). The dataset includes a diverse collection of genomes, each accompanied by essential metadata such as country of isolation and year of collection. Before analysis, all data were subjected to rigorous pre-processing and quality-control measures: after trimming and de novo assembly, any atypical genomes or metagenome-assembled genomes (MAGs) were excluded; assembly completeness and contamination were assessed with CheckM (v1.1.3), and only genomes with ≥ 80% completeness and < 1% contamination were retained. Assemblies were further required to meet the following thresholds: contig N50 > 10 kb, ≤ 500 contigs, total assembly size between 2.0–2.3 Mb, and mean sequencing depth ≥ 30 × . For analyses sensitive to genome length (e.g., core-genome SNP calling, pan-genome analysis), we then narrowed the dataset to genomes with total lengths within 2.1 ± 0.1 Mb. These filtering steps ensured high-quality input for all downstream genomic and AMR analyses.

### 2.2. Identification and validation of antimicrobial resistance genes

AMR-related genes were identified using NCBI’s AMRFinderPlus database (https://www.ncbi.nlm.nih.gov/pathogens/antimicrobial-resistance/AMRFinder/), a command-line tool that utilizes a curated database to predict AMR profiles directly from genomic sequences. WGS data from *N. gonorrhoeae* isolates were analyzed to detect resistance-associated genes against various antibiotics. To ensure accuracy, these predictions were further validated by cross-referencing AST data available in the NCBI Pathogen Detection Database (https://www.ncbi.nlm.nih.gov/pathogens/).

### 2.3. Retrieval of minimum inhibitory concentration data

MIC data were obtained from the NCBI database (https://www.ncbi.nlm.nih.gov/pathogens/ast) for isolates with available whole-genome sequences in the NCBI genome databases. These data were used to assess the correlation between AMR genomic predictions and the phenotypic resistance profiles of the isolates. MIC values, which indicate the lowest antibiotic concentration needed to inhibit bacterial growth, were analyzed across different isolates to identify the resistance patterns.

### 2.4. Multi-locus sequence typing analysis of *Neisseria gonorrhoeae* isolates with PhyloViz

MLST analysis of Neisseria gonorrhoeae isolates was performed using the N. gonorrhoeae MLST scheme available at PubMLST (https://pubmlst.org/bigsdb?db=pubmlst_neisseria_seqdef). Each isolate was characterized by sequencing the seven housekeeping genes required for MLST. The allele profiles were determined using the MLST command-line tool, and STs were assigned based on the allele combinations. Isolates were classified into known STs, and novel STs were identified by comparing allele profiles against the PubMLST database.

For phylogenetic analysis, the STs of the isolates were imported into PhyloViz version 2, a tool used for visualizing and analyzing MLST data. PhyloViz was employed to construct a phylogenetic tree that illustrates the evolutionary relationships among the isolates. The software enabled the clustering of isolates into clonal complexes, providing insights into genetic diversity and clonal expansion within the population. Visualizations generated by PhyloViz facilitated the identification of major genetic lineages and the exploration of the distribution of STs across different geographic regions and periods.

### 2.5. Genomic trends in *Neisseria gonorrhoeae* isolates

To investigate genomic trends in *N. gonorrhoeae*, we integrated each strain’s country of origin and year of isolation. We evaluated the distribution of AMR-related genes and STs across various periods and geographic regions to identify patterns and evolutionary trends. Visualizing the temporal and spatial data allowed us to identify correlations between the emergence of specific resistance profiles and their corresponding isolation times and locations.

### 2.6. Statistical and phylogenetic analysis

All statistical analyses were performed in R (v4.3.0) using the tidyverse, Hmisc and stats packages. Continuous genomic metrics (genome length, total gene count, protein-coding gene count, pseudogene count) were first screened for outliers using the interquartile range (IQR) method (> 1.5 × IQR), and extreme values were excluded. Normality was assessed by the Shapiro–Wilk test:

Pearson’s correlation (Hmisc::rcorr, method = “pearson”) was used to quantify linear associations between normally distributed continuous variables and collection year.

Spearman’s rank correlation (stats::cor.test, method = “spearman”) was applied to non-normal or ordinal data, including annual AMR gene prevalence (%) and MLST frequency trends across years and countries.

Two‐tailed p-values < 0.05 were considered statistically significant. Geographic associations in MLST distributions were further evaluated by chi-square tests.

Phylogenetic relationships were inferred from concatenated MLST loci. Finally, MIC data were integrated to validate genotype–phenotype correlations, with the same correlation framework (Pearson or Spearman) applied depending on variable distributions.

## 3. Result and discussion

In this study, we conducted an in-depth genomic investigation of 38,585 *N. gonorrhoeae* isolates. This study provides critical insights into the genetic architecture, diversity, AMR gene prevalence, and the corresponding phenotypic resistance profiles. The findings are systematically organized to illuminate the complex genomic landscape of this significant human pathogen and trace the evolutionary pathways of AMR.

### 3.1. Global genomic trends in *Neisseria gonorrhoeae* (1990-2025)

The 38,585 *N. gonorrhoeae* WGS dataset from multiple countries exemplifies the increasing reliance on genomic epidemiology for examining AMR and strain diversity [10].

Assembly quality varies, with only a subset of isolates (188 isolates) achieving complete genome assembly, while the majority consists of contigs (38,144 isolates), indicating challenges in genomic studies related to the assembly and management of diversity across regions [11]. Additionally, 31 were assembled at the chromosome level and 222 at the scaffold level. The utility of WGS in addressing AMR and epidemiological questions is supported by numerous studies advocating its critical role in public health strategies and improving treatment protocols [12]. Furthermore, the dataset represents a notable diversity of global strains, reinforcing the interconnected nature of *N. gonorrhoeae* transmission patterns worldwide [13].

The observed variability in assembly quality among *N. gonorrhoeae* isolates reflects the diverse methodologies and sequencing technologies employed across studies. While contig-level assemblies yield informative data, they often present fragmented genomic sequences that complicate subsequent analyses like gene prediction and functional annotation [14]. The average genome size of approximately 2,117,476 base pairs with a modest standard deviation of ±41,042.2 bp aligns with the compact genome structure characteristic of *N. gonorrhoeae* [15]. Notably, an average of 2,236 annotated genes per isolate, including approximately 1,992 protein-coding genes and 191 pseudogenes, indicate an ongoing evolutionary adaptation facilitated by reduced selective pressures and horizontal gene transfer (HGT) [16]. This variability, particularly in the N50 values between contig and scaffold assemblies, emphasizes improved sequencing strategies, such as long-read sequencing technologies, to enhance genome completeness and facilitate accurate functional annotations and resistance gene identification [17].

### 3.2. Evolutionary trends in genomic features across sequence types

**Fig 1:**
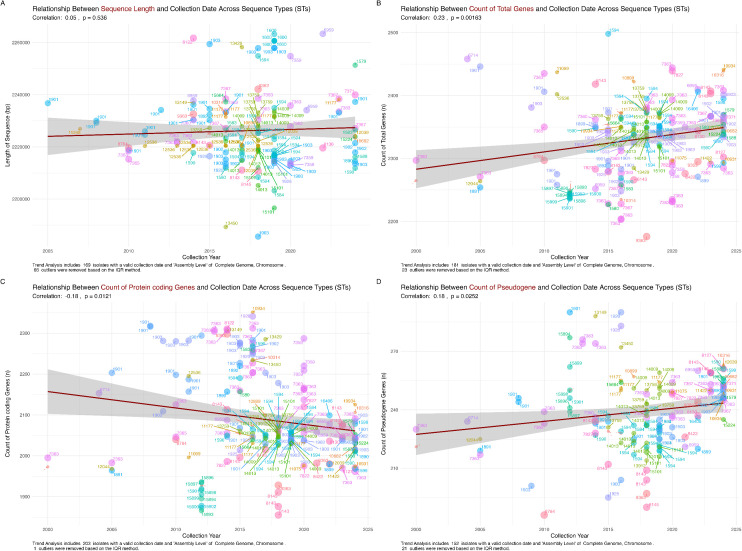
Temporal Analysis of Genomic Features in *Neisseria gonorrhoeae* Across Sequence Types. (A) The relationship between sequence length and collection date across STs shows no significant trend (r = 0.05, p = 0.536), suggesting genome size stability over time. (B) The relationship between total gene count and collection date indicates a significant moderate positive correlation (r = 0.23, p = 0.00163), suggesting an increase in gene count over time, potentially due to HGT or improved annotation. (C) The relationship between protein-coding gene count and collection date shows a weak but significant negative correlation (r = -0.18, p = 0.0121), implying a slight decrease in protein-coding genes over time, possibly due to pseudogenization or gene loss. (D) The relationship between pseudogene count and collection date demonstrates a weak but significant positive correlation (r = 0.18, p = 0.0252), indicating an accumulation of pseudogenes over time, likely reflecting relaxed selective pressures. Each data point represents a unique ST, with color variations distinguishing different STs. The regression lines highlight overall trends while the shaded regions indicate confidence intervals. These findings contribute to understanding *N. gonorrhoeae’s* evolutionary trajectory and adaptation to selective pressures.

To investigate potential evolutionary trends in genomic features, we analyzed the relationship between the collection date across STs and several genomic attributes, including sequence length, total gene count, protein-coding gene count, and pseudogene count (Fig 1).

#### 3.2.1. Relationship between sequence length and collection date across sequence types.

The analysis indicated a statistically non-significant positive correlation (r = 0.05, p = 0.536) between sequence length and collection year for *N. gonorrhoeae* isolates, suggesting remarkable stability in genome size over the past 35 years (Fig 1A). This consistency may indicate that selective pressures, which often impose constraints on genome size, affect this pathogen and optimize replication and resource allocation within the bacterial cell [16]. Despite minor variations in insertions and deletions, the lack of a significant trend in genome size alterations reinforces the notion that changes are sporadic rather than influenced by temporal factors [18]. Such genomic stability is crucial for adapting bacteria to the human host, allowing them to maintain a compact genome while evolving other features, such as gene content, more rapidly in response to environmental challenges, notably antibiotic resistance [3]. The insights drawn from this stability emphasize the evolutionary strategies adopted by *N. gonorrhoeae* to persist and thrive within its ecological niche [19].

#### 3.2.2. Relationship between total gene count and collection date across sequence types.

The statistically significant moderate positive correlation (r = 0.23, p = 0.00163) between the total gene count and collection year in *N. gonorrhoeae* isolates suggests an increasing number of annotated genes over time (Fig 1B). This trend may stem from several factors, such as acquiring novel genes via HGT, gene duplication events, or advancements in annotation methodologies [15]. These gene additions likely represent adaptive evolution, equipping bacteria with advantageous traits to cope with environmental challenges such as antimicrobial exposure and immune responses [20]. Furthermore, improvements in sequencing technologies and bioinformatics methods have enhanced the identification and annotation of these genes, potentially contributing to the observed increase in gene counts [21]. This evolving genetic landscape calls for further exploration to identify the newly acquired genes precisely, elucidate their functional roles, and assess how they enhance the organism’s adaptability, particularly in the context of the rising incidence of AMR [22].

#### 3.2.3. Relationship between protein-coding gene count and collection date across sequence types.

The statistically significant weak negative correlation (r = -0.18, p = 0.0121) between the number of protein-coding genes and the year of collection indicated a slight decrease in protein-coding genes among more recent *N. gonorrhoeae* isolates (Fig 1C). This trend, juxtaposed with an increasing total gene count, suggests a complex interplay between gene gain and loss, potentially involving gene inactivation and pseudogenization processes [23]. Such reductions could be an adaptive response to selective pressures, favoring genome streamlining by eliminating non-essential or redundant genes, thereby enhancing overall genomic efficiency [16]. Interestingly, the decline in protein-coding genes does not necessarily equate to losing functional capacity. This might reflect adaptive evolution in which genes that no longer confer benefits are removed, while the rising total gene count may be due to the incorporation of non-coding RNA genes, regulatory elements, or pseudogenes that play vital roles in gene regulation and adaptability to the host environment [24]. Future investigations should identify specific lost or pseudogenized genes to elucidate their functional implications and connections to *N. gonorrhoeae* adaptation mechanisms related to antibiotic resistance and immune evasion [25].

#### 3.2.4. Relationship between pseudogene count and collection date across sequence types.

The statistically significant weak positive correlation (r = 0.18, p = 0.0252) between pseudogene counts and collection year suggests the accumulation of pseudogenes in *N. gonorrhoeae* isolates over time (Fig 1D). This accumulation may indicate a pseudogenization process, an evolutionary mechanism through which functional genes become inactive due to mutations, often due to relaxed selective pressures or redundancy in gene function [26]. The increase in pseudogenes may signal ongoing genomic decay or functional adaptation via gene inactivation, potentially offering selective advantages in changing environments [27]. Pseudogenes can play crucial regulatory roles by acting as reservoirs of genetic diversity, which may enable rapid responses to environmental challenges such as antibiotic exposure [28]. This regulatory potential of pseudogenes highlights their importance as certain mutations may regain relevance when organisms face new selective pressures [29]. Investigating specific pseudogenes that accumulate over time will enhance our understanding of their origins, persistence factors, and functional contributions to the adaptability and survival of *N. gonorrhoeae* [26].

### 3.3. Distribution of sequence types over time

**Fig 2:**
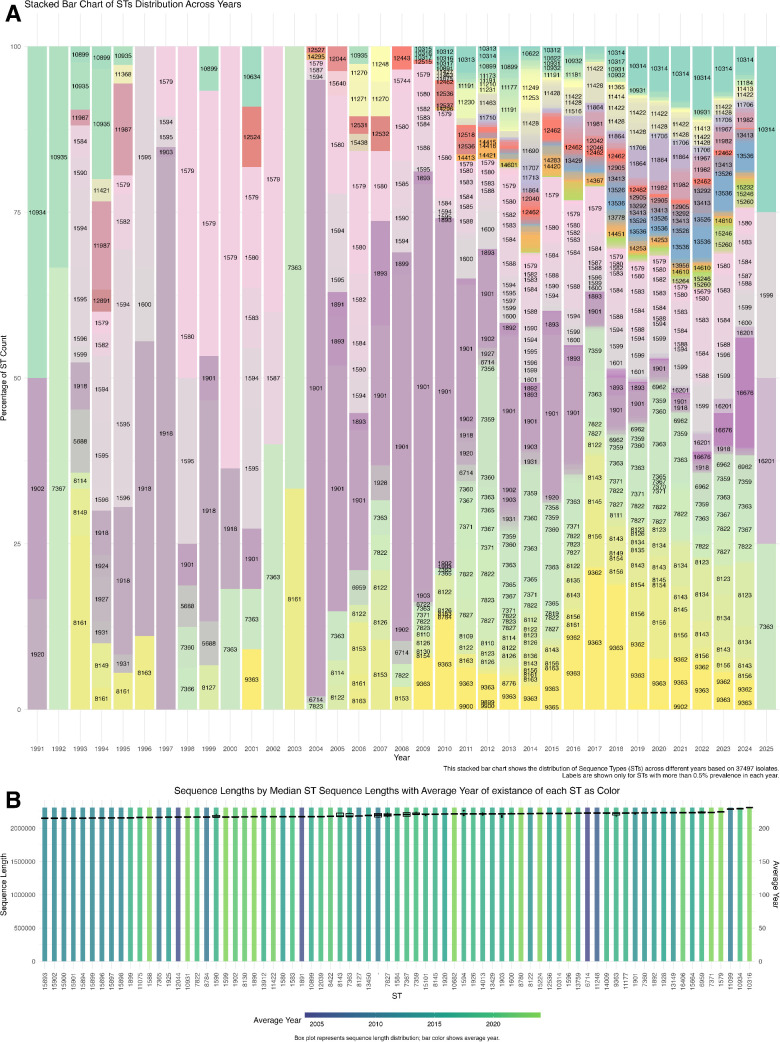
Temporal Trends in Sequence Type Distribution and Genome Length Variation in *Neisseria gonorrhoeae.* Temporal trends in sequence type distribution and genome length variation in Neisseria gonorrhoeae. (A) Stacked bar chart of dominant STs (≥ 0.5% prevalence) from 1990 to 2025 (n = 37 471 isolates), highlighting shifts and persistence of ST-1901, ST-1600 and ST-1579. (B) Box plot of sequence length for each ST, colored by mean year of isolation, showing stable median lengths despite evolutionary pressures.

We analyzed ST distribution to explore N. gonorrhoeae population structure over time. Fig 2A shows the prevalence of dominant STs from 1990 to 2025, highlighting ST-1579, ST-1600, and ST-1901’s persistence. These STs fluctuated in relative abundance, suggesting ongoing population shifts and clonal expansion. Their sustained prevalence indicates strong adaptive potential, likely due to ecological factors like antibiotic exposure and host immune responses [30].

ST-1579, ST-1600, and ST-1901 persistence in *N. gonorrhoeae* suggest adaptive mechanisms to resist antibiotic exposure and host immune responses. Alterations in *PenA*, conferring beta-lactam resistance, occur via HGT from commensal Neisseria species, contributing to resistant strains [31]. The Gonococcal Genetic Island (GGI) defines distinct subpopulations of N. gonorrhoeae, potentially enabling them to acquire traits that enhance survival against host immune mechanisms [32]. The high frequency of HGT among N. gonorrhoeae populations facilitates rapid antibiotic adaptation, highlighting the ongoing evolutionary pressure of antibiotic use in clinical settings [33]. Genetic adaptability in pathogenic N. gonorrhoeae allows it to thrive in hostile environments. ST-1901 and ST-1600 exhibit significant antibiotic resistance, posing public health concerns. ST-1901 is particularly resistant to extended-spectrum cephalosporins and macrolides due to genetic adaptations, including mutations in key resistance genes like *penA*, *mtrR*, and *gyrA* [34]. These mutations contribute to global clonal dissemination and the persistence of resistant strains, underscoring the urgency for robust monitoring systems and targeted interventions [35]. Similarly, ST-1600 has reduced susceptibility to multiple antibiotics, indicating that genetic factors promote resistance and enhance survival under selective pressures [34]. The improved fitness associated with these strains suggests that resistance determinants can be acquired and spread among bacterial populations, further complicating the treatment options [35]. The necessity for comprehensive genomic surveillance is thus highlighted, emphasizing the need to track chromosomal and plasmid-mediated resistance mechanisms to inform public health strategies [36].

Fluctuations in ST abundance in *N. gonorrhoeae* complicate its epidemiology, with antibiotic treatment and host immune responses key. ST-1901’s increasing prevalence reflects its global prevalence, highlighting the emerging threat of resistance, especially to third-generation cephalosporins [37]. Conversely, ST-1579 and ST-1600 displayed stability despite the emergence of resistant clones, suggesting a nuanced interaction between these strains and treatment practices [38,39]. Continuous genomic surveillance is crucial for monitoring evolutionary dynamics and adapting treatment protocols [40]. Despite resistance, understanding genetic shifts in N. gonorrhoeae populations is crucial for effective AMR strategies and treatment guidelines [41].

### 3.4. Temporal stability of genome size within specific sequence types

Fig 2B shows the temporal stability of genome size within specific STs. The median sequence length is plotted against the average year of isolation. Isolates within specific STs maintained relatively stable genome sizes. Slight variations were observed across STs, suggesting genome size is a characteristic feature of specific N. gonorrhoeae lineages. Some STs increased median sequence length, indicating ongoing genome evolution and potential adaptive changes. These increases may be due to acquiring specific genomic islands or mobile genetic elements, contributing to lineage-specific adaptations.

The stability in genome size among certain STs of *N. gonorrhoeae* suggests a strong evolutionary constraint. Conversely, the increase in genome size for other STs likely reflects the integration of mobile genetic elements, such as plasmids or genomic islands, which confer selective advantages by acquiring genes linked to virulence and antibiotic resistance. Plasmids are crucial for the horizontal transfer of resistance determinants, enhancing bacterial survival in diverse environments [42]. The CRISPR-Cas system in many bacteria captures and integrates genetic material from mobile elements, supporting *N. gonorrhoeae’s* adaptation and evolutionary dynamics under antibiotic pressure and host immune responses [43]. The ongoing genomic adaptations in specific STs show *N. gonorrhoeae’s* evolutionary trajectory in response to external pressure.

### 3.5. Tree-map visualization of st distribution across countries

**Fig 3:**
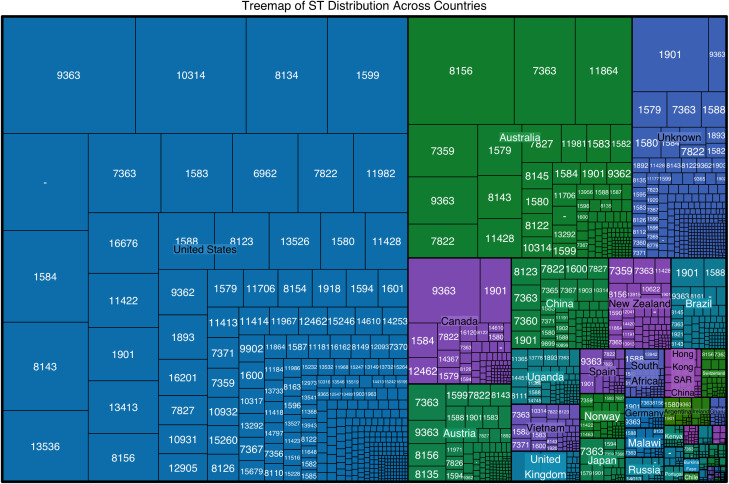
Treemap of *Neisseria gonorrhoeae* sequence type distribution across countries. Each rectangle represents an ST, sized by its frequency and colored by geographic region. Darker shading indicates higher prevalence. ST-9363, ST-1579 and ST-1901 dominate in the United States, Canada and Australia, illustrating regional population structure.

The global distribution of N. gonorrhoeae STs is illustrated in a tree-map (Fig 3). Each rectangle represents a unique ST, with size reflecting its frequency in the dataset. This facilitates visual comparison of ST prevalence by location. The color gradient encodes the relative frequency of each ST within countries, with darker shades indicating higher prevalence [44]. This approach effectively highlights the diversity of STs and patterns that may inform targeted public health strategies, especially in regions reporting rising AMR [45]. Overall, the tree-map. It is a powerful tool for epidemiological analysis, contributing to our understanding of the global dynamics surrounding *N. gonorrhoeae* transmission and resistance patterns [46].

The tree-map visualization of *N. gonorrhoeae* STs shows a non-uniform distribution across countries, reflecting geographical structuring in the population. The US and Australia have larger blocks for prevalent STs, like ST-1901 and ST-7363. ST-1901 is particularly prevalent in Australia, while ST-1594 is highly prevalent in the United States [23]. This pattern shows significant regional variations in the distribution of dominant STs, suggesting adaptation or origins specific to these areas. Context-aware public health strategies are needed to address AMR challenges in these regions [47].

The geographical clustering of STs in N. gonorrhoeae highlights the importance of regional epidemiology in understanding population dynamics and AMR patterns. Local antibiotic usage and healthcare practices likely influence the persistence and dissemination of specific STs across regions [48,49]. The dominance of STs like ST-1594 in the US and ST-1901 in Australia and Canada indicates that localized antibiotic resistance profiles can shape clone success [49]. Increased antibiotic usage and selective prescription practices in some countries may favor resistant STs, complicating treatment [50]. Local outbreaks and sexual network dynamics persist and spread certain STs, while migration and travel patterns influence the geographic distribution of specific clones [51]. Such insights necessitate a tailored approach for public health strategies and interventions targeting *N. gonorrhoeae*, accounting for the unique epidemiological landscapes of different regions.

The non-uniform distribution of *N. gonorrhoeae* STs emphasizes the critical need for global surveillance programs that monitor the spread of dominant clones and effectively track resistance trends. Surveillance is essential for identifying emerging resistance patterns and facilitating timely public health intervention [52]. Localized clusters may signify regional outbreaks or the evolution of STs within isolated populations where gene flow is limited [53]. Reduced genetic diversity can contribute to local adaptations or the persistence of specific STs due to localized environmental pressures and treatment regimens [54]. To control outbreaks and prevent resistant clone spread, targeted regional strategies and global monitoring systems are essential. Understanding the interaction between healthcare practices, antibiotic use, and ST dynamics is crucial for effective interventions against *N. gonorrhoeae* and its resistance [54,55].

### 3.6. Minimum spanning tree analysis of genetic diversity

**Fig 4:**
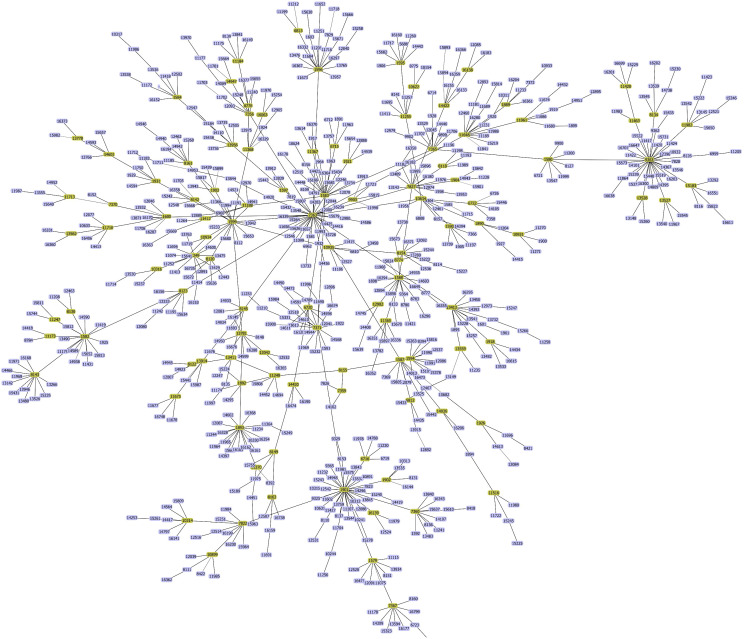
Minimum spanning tree of *Neisseria gonorrhoeae* core-genome diversity. Network of 109 clades (yellow circles) based on core SNPs. Circle size reflects number of isolates; edge length corresponds to genetic distance. Major clonal complexes form densely connected clusters, while divergent lineages appear as longer branches.

A core-genome SNP–based minimum spanning tree (MST) of 38 585 isolates resolved into 109 distinct clades (Fig 4), with each node sized by isolate count and edge length reflecting genetic distance [56]. Dominant STs—such as ST-1901 and ST-1579—formed tight, densely connected clusters, whereas longer branches marked more divergent lineages, likely representing older or regionally isolated populations [57]. The occurrence of the same ST in multiple clades underscores frequent recombination and evolutionary connectivity among lineages. This structured network confirms a complex clonal architecture in N. gonorrhoeae and demonstrates the MST’s utility for high-resolution tracking of emerging and spreading resistant clones [32,56–58].

### 3.7. Gene prevalence across *Neisseria gonorrhoeae* isolates and antimicrobial resistance gene families

Analysis of the AMR gene landscape of *N. gonorrhoeae* has revealed critical insights into the genetic mechanisms underlying resistance. The prevalence of certain AMR genes, such as *norM* (100%), *farB* (99.99%), and *mtrC* (100%), indicates that they are core components of the *N. gonorrhoeae* genome. These efflux pump genes are essential for resistance against various antimicrobial agents, highlighting their potential roles beyond AMR, such as mediating stress responses or aiding in adaptation to host immune pressures [59]. The ubiquitous presence of these efflux pumps mirrors the findings in other pathogenic bacteria, where similar systems contribute to antibiotic resistance and overall virulence and survival in host environments [60]. This suggests that *N. gonorrhoeae* utilizes these pumps to endure antimicrobial pressure while responding to other environmental stresses, including immune system attacks and nutrient limitations [61]. The broad distribution of efflux genes among isolates, particularly regarding global patterns of antibiotic use, reinforces the significance of continued surveillance of emerging resistance mechanisms in *N. gonorrhoeae* [62]. Understanding these gene dynamics is vital for developing effective clinical strategies to combat resistance and manage gonorrhea infections.

The detection of *bla*_TEM-1_, which confers beta-lactam resistance in *N. gonorrhoeae* isolates, reflects a notable challenge concerning antibiotic efficacy. This prevalence illustrates the ongoing burden of beta-lactamase-mediated resistance, echoing the findings from global studies documenting similar resistance patterns [63]. While genetic determinants such as the *bla*_TEM_ gene indicate resistance, the frequencies of these genes compared to efflux pump genes may mitigate immediate treatment concerns yet underscore the potential for treatment failure [64]. Moreover, the historical reliance on beta-lactams, particularly penicillin, makes this persistence alarming, indicating an adaptive response of *N. gonorrhoeae* to antibiotic pressure [65]. Continuous monitoring of resistance mechanisms, including those attributed to beta-lactamases, is crucial for effectively managing gonococcal infections and mitigating treatment failures associated with declining antibiotic effectiveness [66].

The analysis of antibiotic resistance genes in *N. gonorrhoeae* highlights notable genes like *bla*_TEM-135_ (3.08%) and *bla*_TEM-239_ (0.32%), contributing to beta-lactam resistance. Although these genes are present at lower frequencies, their existence indicates a diversity of β-lactamase genes circulating within *N. gonorrhoeae* populations. This genetic diversity may respond to selective pressures stemming from the widespread use of β-lactam antibiotics [63]. The low prevalence of these genes might reflect regional variations in antibiotic usage or signify nascent stages of dissemination within certain isolates or populations. Identifying these rare variants may also provide insights into potential future resistance patterns, as plasmid-mediated β-lactamases suggest an avenue for HGT, enhancing resistance among different bacterial strains [65]. Epidemiological studies have reported that the emergence of extended-spectrum beta-lactamases (ESBLs) within *N. gonorrhoeae* is concerning because of their potential to confer resistance to penicillins and cephalosporins, which remain critical for gonorrhea treatment [67]. Monitoring these rare β-lactamase genes is crucial for understanding the evolving resistance patterns that could pose significant public health challenges in the future [68].

The presence of genes such as *bla*_TEM-245_, *bla*_TEM-186_, and *erm(F)* in less than 0.01% of *N. gonorrhoeae* isolates suggests minimal roles in AMR. However, these low-frequency genes often reside on plasmids or transposons, making them primed for horizontal transfer and rapid expansion if selective pressures change [69]. For example, a single introduction of *erm(F)* into a high-transmission lineage could quickly undermine macrolide efficacy. Consequently, monitoring these rare variants provides an early warning system for emergent resistance phenotypes, especially in regions with intensive antibiotic use or disinfectant exposure [63,70]. Incorporating sentinel surveillance for low-prevalence AMR genes will help public health authorities detect and contain nascent resistance threats before they become widespread.

### 3.8. Prevalence and global distribution of antimicrobial resistance gene families in *Neisseria gonorrhoeae*

**Fig 5:**
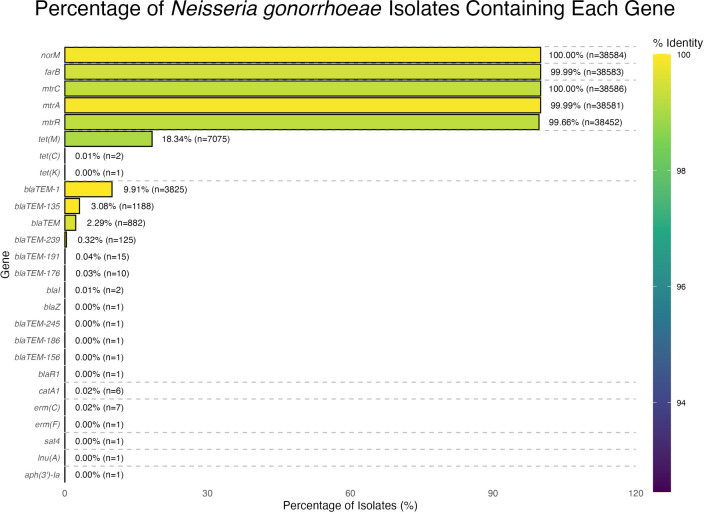
Prevalence of antibiotic resistance–associated genes in *Neisseria gonorrhoeae* isolates. Bar chart showing percentage of isolates carrying each gene. Core efflux pump genes (*norM*, *farB*, *mtrC*, *mtrA*, *mtrR*) approach 100% prevalence; *tet(M)* is present in 18.3% of isolates and *bla*_TEM*-1*_ in 9.9%. Color intensity indicates percent identity to reference sequences; dashed lines mark genes detected at very low frequency (≤ 0.01%).

**Fig 6:**
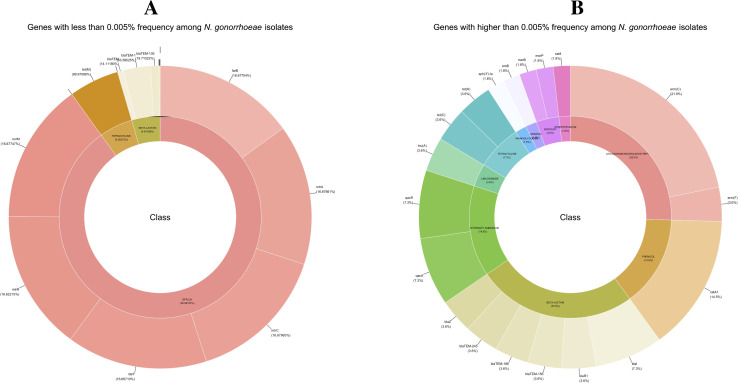
Gene frequency distribution across *Neisseria gonorrhoeae* isolates. (A) Low-frequency AMR genes (< 0.005%), grouped by class (beta-lactam, tetracycline, efflux). (B) High-frequency AMR genes (> 0.005%), highlighting *bla*_TEM*-1*_*, tet(C)*, *erm(C)* and *norM*. Colors denote antimicrobial classes.

Building on the overall distribution of resistance-associated genes shown in Fig 5, our analysis of AMR gene families in *N. gonorrhoeae* has identified two distinct patterns based on gene prevalence. Low-frequency genes (<0.005% of isolates) such as *bla*_TEM_, *tet*(M), *mtrF*, and *mtrR* appeared to play a limited role in resistance (Fig 6A). In contrast, highly prevalent AMR genes (>0.005%), including *bla*_TEM-1_, *tet*(C), *erm*(C), and *norM*, significantly contributed to beta-lactam and tetracycline resistance (Fig 6B). The widespread presence of these genes suggests intense selective pressure driving resistance. Additionally, the *qac* gene family (*qacR* and *qacA*), associated with resistance to quaternary ammonium compounds used in disinfectants, has been identified across diverse geographic regions, highlighting the potential role of environmental factors in AMR dissemination [4].

Several other AMR gene families further illustrate the complex resistance landscape of *N. gonorrhoeae*. The *lnu*(A) gene confers lincosamide resistance. It is widely distributed throughout North America, Europe, and Africa [71,72]. Similarly, *sat4*, linked to streptothricin resistance, is found in isolates from Russia, Canada, and the U.S., emphasizing the impact of agricultural antibiotic use on human pathogens [73–75]. The *erm* gene family, including *erm*(A), *erm*(B), and *erm*(C), mediates resistance to macrolides and lincosamides, raising concerns about cross-resistance and the role of both clinical and agricultural antibiotic use [76–79]. Tetracycline resistance genes, including *tet*(A), *tet*(B), and *tet*(C), are present globally, reinforcing the impact of antibiotic overuse in medical and veterinary settings [80,81]. Beta-lactam resistance, mainly associated with *bla*_TEM_, *bla*_SHV_, and *bla*_CTX-M_, signifies the need for ongoing genomic surveillance to monitor the evolution [82–84]. Finally, aminoglycoside resistance genes (*aac*) have been detected across multiple STs and regions, further supporting the need for continuous surveillance and stewardship efforts to mitigate the impact of AMR on *N. gonorrhoeae* [85,86].

### 3.9. Geographical distribution of antimicrobial resistance genes in *Neisseria gonorrhoeae*

**Fig 7:**
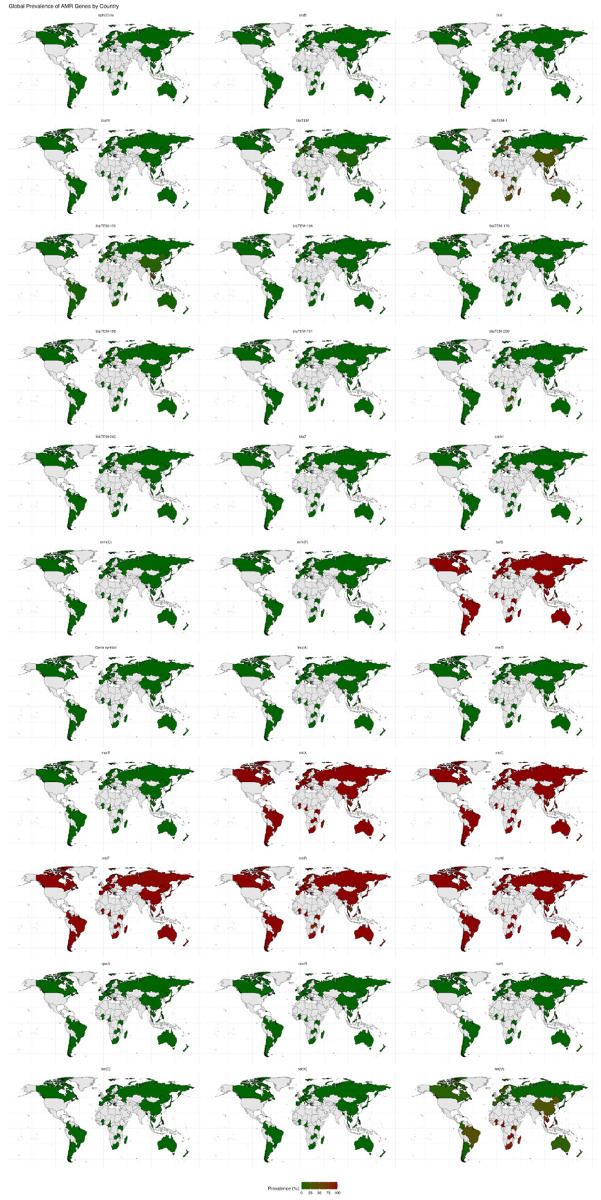
Global prevalence of antimicrobial resistance genes by country. Choropleth maps for key genes (*mtrC*, *erm(C)*, *bla*_TEM_, *tet(C)*, *norM*), with darker shading indicating higher prevalence. Regional patterns reflect local antibiotic usage and stewardship practices. Global map visualization created using OpenStreetMap data (https://www.openstreetmap.org), available under the Open Database License (ODbL). Map data OpenStreetMap contributors, licensed under ODbL.

The Global Prevalence of AMR Genes by Country is presented in Fig 7. The geographical distribution of AMR genes in *N. gonorrhoeae* revealed notable trends that reflected regional antibiotic usage and public health practices. Core resistance genes such as *norM*, *mtrC*, and *tet*(M) have established a high prevalence across continents, particularly in the United States and Europe, indicating widespread adaptation to antimicrobial pressures [87]. This adaptability suggests evolutionary advantages conferred by core resistance mechanisms, allowing *N. gonorrhoeae* to thrive in various environments and potentially evade host defense strategies [65]. In contrast, the *erm*(C) gene, which confers resistance to macrolides, is more localized, with higher prevalence in Europe and Asia. This regional concentration likely correlates with the intensive use of macrolides in treating respiratory infections, demonstrating how local prescribing habits influence the resistance landscape [88]. The differential prevalence of *bla*_TEM_ and *aac* genes further emphasizes geographical variability in resistance profiles due to local healthcare infrastructures and antibiotic policies [89]. Monitoring these geographical trends through specialized surveillance programs is crucial for tailoring public health interventions and adapting treatment protocols, effectively managing AMR as it evolves. Such region-specific strategies are essential for capturing emerging resistance patterns that could threaten global health security [90].

### 3.10. Temporal trends in antimicrobial resistance gene accumulation in *Neisseria gonorrhoeae*

**Fig 8:**
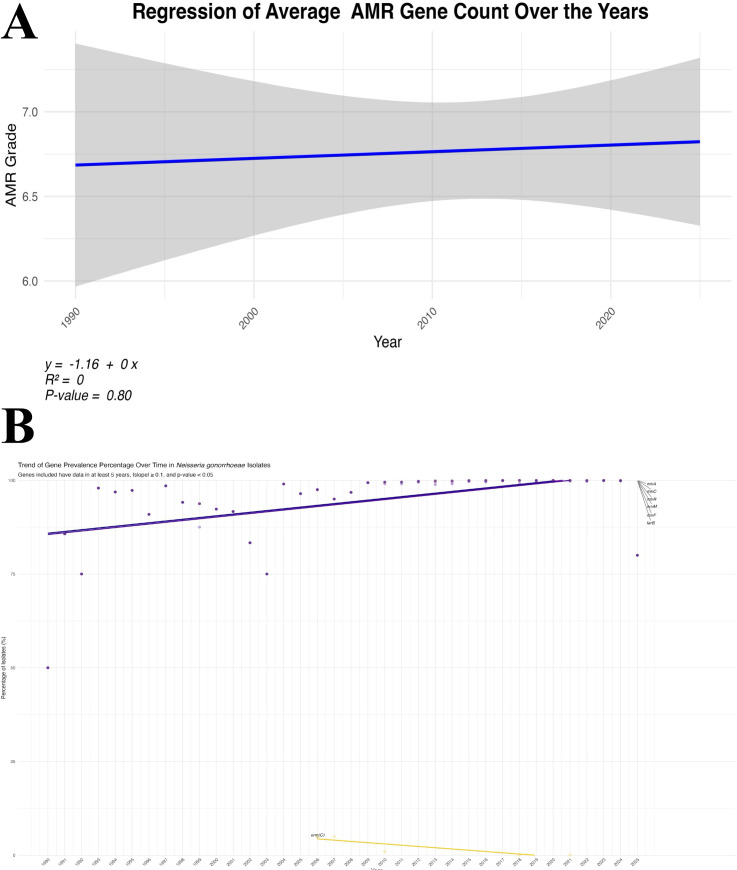
Trend analysis of AMR gene prevalence over time in *Neisseria gonorrhoeae* isolates. (A) Regression of mean AMR gene count per isolate (R² = 0.80, p < 0.05), showing a gradual increase from 1990 to 2025. (B) Line plots of individual gene prevalence over time, with rising genes (purple lines) and declining genes (e.g., *erm(C)*).

To investigate temporal trends in the accumulation of AMR genes, we performed a regression analysis of the average AMR gene count per isolate over the collection years (Fig 8A). The regression analysis examines the relationship between the average number of AMR genes detected in isolates from each year and the corresponding collection year.

The regression analysis, depicted in Fig 8B, demonstrates a slight but statistically significant upward trend in the average AMR gene count over time.

The regression line shows a gradual increase in AMR gene burden across the decades, with a coefficient of determination (R²) value of 0.80. The analysis of temporal trends in the accumulation of AMR genes in *N. gonorrhoeae* reveals a statistically significant upward trend over the years studied, represented by an R² value of 0.80. This strong correlation suggests that the population of *N. gonorrhoeae* has consistently accumulated AMR genes, possibly due to increased selective pressure from antibiotic usage [54]. The gradual rise in AMR gene counts indicates the bacteria’s ability to acquire and maintain resistance mechanisms, which has profound implications for treatment and public health strategies [91].

Furthermore, the upward trend aligns with the emergence of specific resistance genes such as *norM* and *mtrC*, which are well-established efflux pumps contributing to MDR. Their proliferation among isolates hints at the evolutionary advantages these genes confer under antibiotic pressure [92]. However, the relatively shallow slope of the regression line implies that while resistance is accumulating, it is not accelerating rapidly; changes in treatment protocols could be moderating the pace at which resistance evolves [93]. For instance, the observed decrease in *erm*(C) prevalence suggests successful stewardship in certain regions, hinting that targeted interventions can effectively influence resistance patterns [94].

Continued monitoring of AMR gene trends in *N. gonorrhoeae* is essential for informing effective public health interventions and adjusting treatment guidelines to counteract the threat of rising resistance.

### 3.11. Antibiotic susceptibility and minimum inhibitory concentrations trends in *Neisseria Gonorrhoeae*

The analysis of MICs for *N. gonorrhoeae* provides critical insights into the antibiotic susceptibility landscape. Our study revealed significant variations in susceptibility across various antibiotics compared to the resistance breakpoints established by the Clinical and Laboratory Standards Institute (CLSI). The average MIC values for antibiotics such as ceftriaxone and azithromycin highlighted concerning trends of increasing resistance among isolates over time [95]. High-level resistance to ceftriaxone has become problematic, as observed in strains associated with various STs, including those demonstrating a novel *PenA* allele [37].

**Fig 9:**
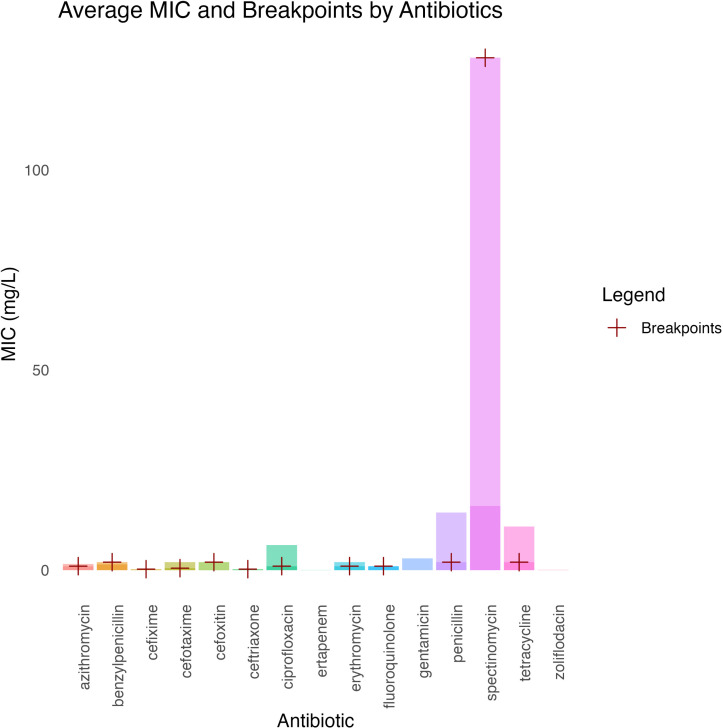
Comparison of average minimum inhibitory concentration values and clinical breakpoints across antibiotics in *Neisseria gonorrhoeae.* Dot plot of mean MICs for key drugs; red crosses mark CLSI resistance breakpoints. Penicillin and spectinomycin exceed breakpoints, while azithromycin, ceftriaxone and ciprofloxacin remain below.

Fig 9 presents the average MIC values for various antibiotics, with red cross markers indicating the corresponding resistance breakpoints. The gradual increase in MICs for critical antibiotics reflects a worrying trajectory, as evidenced by a consistent elevation in resistance patterns among gonococcal strains [96]. For instance, studies in Canada demonstrated rising MICs for this pathogen, indicating a pressing need for new treatment strategies [96]. This evolving profile of *N. gonorrhoeae* underscores the importance of robust surveillance programs and the need to evaluate susceptibility patterns to guide clinical decisions and inform public health initiatives to tackle AMR effectively [97].

#### 3.11.1. High resistance patterns in *Neisseria gonorrhoeae.*

The penicillin and spectinomycin resistance data in *N. gonorrhoeae* indicate trends in MIC values exceeding the CLSI resistance breakpoints. Widespread resistance to penicillin is associated with mutations in the *PenA* gene, which are commonly linked to beta-lactam resistance [98]. Consequently, the ineffectiveness of penicillin as a treatment option for gonococcal infections is becoming a significant global concern, reflected in the increasing reports of treatment failures. Spectinomycin resistance has also been documented, particularly in various STs among Russian isolates [99]. Reports indicate that reliance on spectinomycin is diminishing, especially as more strains reach or exceed the resistance thresholds, complicating treatment protocols for gonorrhea. While spectinomycin has historically been an alternative treatment, the emergence of high MIC values indicates that its efficacy is waning, especially among resistant strains [100]. This situation necessitates the development of alternative treatment options and implementing robust antimicrobial stewardship programs to manage and mitigate the rising rates of resistance in *N. gonorrhoeae* [101].

#### 3.11.2. Moderate resistance patterns in *Neisseria gonorrhoeae.*

The assessment of antibiotic susceptibility in *N. gonorrhoeae* revealed that several antibiotics, including azithromycin, cefixime, cefotaxime, ceftriaxone, ciprofloxacin, and tetracycline, exhibited varying levels of resistance. Recent studies indicate the prevalence of resistance to azithromycin, ceftriaxone, and ciprofloxacin has been particularly concerning, with some strains exhibiting resistance levels above CLSI breakpoints [102]. Notably, ciprofloxacin and tetracycline demonstrated elevated MICs, highlighting a troubling trend of increasing resistance to these critical antibiotics, which have historically been vital for treating *N. gonorrhoeae* infections [102]. The implications of this resistance are substantial, complicating treatment regimens and necessitating the more frequent use of third-generation cephalosporins and the development of new therapeutic strategies [103]. The recognition of fluoroquinolone and azithromycin resistance is particularly alarming, given their previous utility in clinical management and the potential for increased treatment failures [102]. These trends underscore the urgent need for robust antimicrobial stewardship programs and enhanced surveillance to combat the evolving resistance landscape in *N. gonorrhoeae* [103].

#### 3.11.3. Low resistance patterns in *Neisseria gonorrhoeae.*

Fluoroquinolones have shown variable susceptibility trends in evaluating AMR in *N. gonorrhoeae* isolates. Some studies indicate that certain fluoroquinolones, such as sitafloxacin, exhibit lower MICs against resistant *N. gonorrhoeae* isolates. However, the overall resistance levels have been rising, challenging their efficacy [104]. Continuous monitoring remains crucial to detect emerging resistance trends. Given the historical difficulties with other antibiotics, carefully managing fluoroquinolone use is imperative to mitigate potential resistance development [105]. Resistance analysis indicates a critical challenge, particularly high-level resistance to penicillin and azithromycin, alongside increased resistance in other classes [55]. While some fluoroquinolones may still be effective, the increasing resistance to penicillin, azithromycin, and cephalosporins complicates treatment strategies [106]. Ongoing surveillance of antibiotic resistance trends is essential in shaping effective treatment guidelines and incorporating novel therapeutic strategies for managing MDR *N. gonorrhoeae* infections. Proactive measures are key to sustaining the clinical utility of existing antimicrobials while addressing the ongoing threat of resistance [107].

### 3.12. Correlation between AMR gene presence patterns and phenotypic resistance in *Neisseria gonorrhoeae*

**Fig 10:**
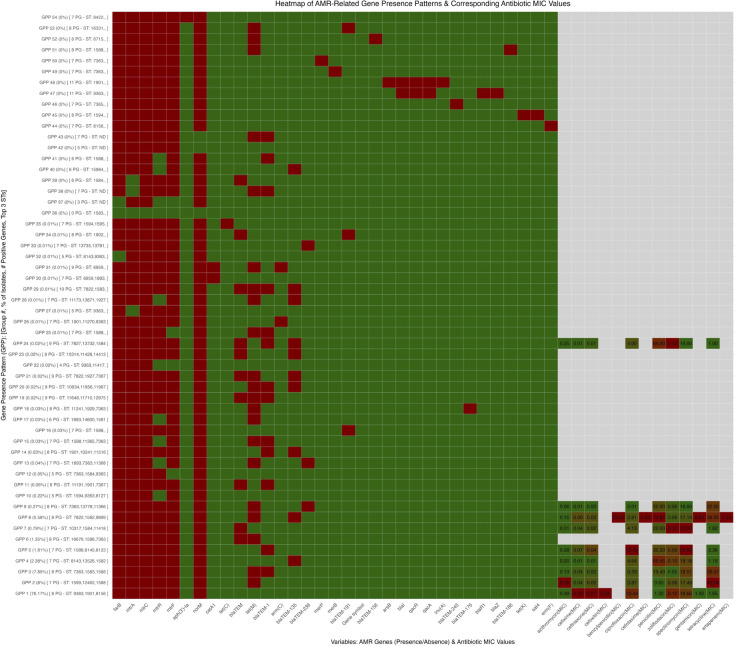
Heatmap of AMR gene presence patterns and corresponding antibiotic MICs in *Neisseria gonorrhoeae.* Rows represent gene presence patterns; columns represent AMR genes (green = present, red = absent). The right panel shows MIC values for each pattern (higher values = greater resistance), illustrating genotype–phenotype correlations.

We generated a heatmap to explore the relationship between specific AMR gene presence patterns (GPPs) and phenotypic antibiotic resistance (Fig 10). The analysis of AMR GPPs in *N. gonorrhoeae* reveals a complex interplay between genetic determinants and phenotypic antibiotic resistance, as illustrated by generated heatmaps. The efflux pump genes, including *mtrC*, *farB*, *norM*, and *mtrA*, exhibit widespread prevalence across multiple GPPs, highlighting their critical role in MDR. These genes facilitate the expulsion of antibiotics, diminishing their intracellular efficacy and enhancing bacterial survival in adverse conditions [108]. Additionally, genes associated with tetracycline resistance such as *tet*(C) and aminoglycoside resistance genes like *aph* (3*’*)-*Ia* are present across various GPPs but are generally less ubiquitous than efflux pump genes, suggesting that while tetracycline resistance is notable, efflux mechanisms may serve as more universally adaptive strategies for survival against diverse antimicrobial stresses [109]. The relatively high MIC values for penicillin highlight significant resistance trends linked to beta-lactamase genes (e.g., *bla*_TEM_), contributing to the diminishing effectiveness of this treatment [110]. Similar trends are observed for spectinomycin, indicating the erosion of options for addressing gonococcal infections and emphasizing the urgent need for comprehensive stewardship and surveillance programs [39]. Conversely, lower MIC values for newer agents like zoliflodacin and gentamicin suggest these treatments may still be viable against resistant *N. gonorrhoeae* strains. However, consistent monitoring is vital as overreliance on these antibiotics could precipitate resistance development [111]. The findings from this study underscore the importance of implementing localized antibiotic stewardship programs and enhancing surveillance systems to identify and respond to emerging resistance patterns in *N. gonorrhoeae* preemptively.

### 3.13. Limitations

Our study relies exclusively on publicly available whole‐genome sequences and accompanying metadata, which vary in quality and completeness. Geographic and temporal sampling biases—driven by differing sequencing efforts across regions and time—may skew observed ST and AMR gene distributions. Incomplete metadata (e.g., missing isolation source, clinical context or MIC values) further limits stratified analyses. Although we applied stringent quality‐control thresholds, residual assembly artifacts or uneven read depths could influence gene detection sensitivity. Finally, our use of AMRFinderPlus and existing resistance‐gene catalogs constrains detection to known sequence variants; novel mechanisms (e.g., promoter mutations, structural rearrangements or entirely new genes) would escape our pipeline. Additionally, we did not perform a core‐genome MLST (cgMLST) or core‐genome SNP analysis, nor did we integrate NG-STAR allele profiles, both of which would provide higher resolution of strain relatedness and AMR‐allele clustering and are recommended for future studies. Future work incorporating targeted long‐read sequencing, functional assays and continuous database expansion will help capture these emerging resistance determinants.

## 4. Conclusion

This study provides a large-scale genomic analysis of *Neisseria gonorrhoeae*, examining AMR gene prevalence, phenotypic resistance, and evolutionary trends. The findings reveal genomic stability over time but a gradual increase in AMR gene burden due to HGT and antibiotic pressure. Key resistance determinants are crucial in MDR, including efflux pumps (*mtrC, farB, norM*) and beta-lactamase (*bla*_TEM_) genes. The analysis of MICs confirms widespread resistance to penicillin and spectinomycin, while newer agents like zoliflodacin and gentamicin remain effective but require monitoring. Dominant STs (ST-1901, ST-1600) persist globally, indicating strong adaptive potential. The study highlights regional variations in AMR gene distribution, emphasizing the need for targeted antibiotic stewardship and surveillance.

Overall, the study underscores the urgent need for improved monitoring strategies, enhanced sequencing techniques, and alternative treatment options to manage the growing threat of antibiotic resistance in *N. gonorrhoeae*.

### Ethics statement

This study did not involve direct human or animal subjects. All data analyzed were anonymized and retrieved from publicly accessible repositories, following ethical guidelines for the use of secondary data. Ethical approval was not required for this research.

## Supporting information

S1 File Comprehensive Supplementary Table: A Synopsis of All Data Analyzed in This Study. (CSV)
